# Psychosocial well-being in Long-Term Care in the Wake of COVID-19: Findings from a Qualitative Study in New Zealand

**DOI:** 10.1007/s10823-023-09485-3

**Published:** 2023-07-19

**Authors:** Rosemary Frey, Deborah Balmer

**Affiliations:** 1 School of Nursing, Faculty of Medical and Health Sciences, University of Auckland, 85 Park Road, Grafton, Auckland, New Zealand; 2 Western Australian Centre for Rural Health (WACRH), University of Western Australia, Geraldton, Australia

**Keywords:** Covid-19, Well-Being, Aged Residential Care, Whare Tapa Whā

## Abstract

Drawing on Mason Durie’s (1985) New Zealand Whare Tapa Whā model of health (spiritual, emotional, physical, and family domains), the goal was to link a model of well-being with the lived reality for long-term care residents and bereaved family members during COVID-19. Interviews were conducted with five residents and six family members of previous residents of one long-term care in one urban centre between July and September 2020. The increased demands imposed by the pandemic highlighted the gaps in well-being for residents and families. In particular, the inability to connect with family during COVID-19 restrictions reduced perceptions of well-being for residents. Study findings indicate that the provision of well-being for older adults and families in long-term care extends beyond the narrow bounds of the biomedical model. The Whare Tapa Whā model provides a valuable framework describing the holistic balance needed between the four health domains.

Studies have reported that health satisfaction is a key component of life satisfaction in later life (Hutchinson & Warner, 2015; Kööts–Ausmees & Realo, 2015). However, according to Wiliyanarti et al. (2020, p. 21), health is a “perfect state that includes physical, mental, social, and spiritual well-being. It does not mean being free from disease only.“ Indeed, while later life is associated with chronic health problems and disabilities, older adults’ subjective views of their health are often positive (Idler, 1993). Accordingly, recent studies explore the aging process framed as positive aging (Hill & Smith, 2015).

Well-being is a multidimensional construct, including subjective, psychological, spiritual, and social dimensions (Wiliyanarti et al., 2020). Subjective well-being can be defined as the happiness and satisfaction a person attains in their life, which is related to the body, mind, and context in which they live (Diener et al., 2009). Spiritual and religious belief has been frequently reported as playing an integral role in the well-being of older adults (Cohen & Koenig, 2003; Koenig, 2012; Malone & Dadswell, 2018). Spirituality can provide an important coping mechanism (Diener et al., 2011). Specifically, a person’s health can be influenced by negative or positive religious/spiritual coping mechanisms (Pargament et al., 1998). Dimensions of psychological well-being and integration with social networks may also play a role in explaining these positive self-evaluations (Guindon & Cappeliez, 2010). Decades of research have demonstrated the link between social network support and both physical and psychological well-being (Cohen, 2004; Goldsmith & Albrecht, 2011; Seeman & Berkman, 1988; Thoits, 2011; Wright, 2016).

*Well-being in Long Term Care (LTC)*. Both structure and culture shape residents’ lives within LTC facilities (Frey et al., 2015). According to Tuckett (2010, p. 2605), LTC can be characterised as ‘promoting instrumental care, prioritising “doing for” over “being with” isolating residents in an environment that is “far from homelike.“ Increased dependency, instrumental care, and challenges to a sense of self and identity may increase the risk of loneliness in LTC residents (Neves et al., 2019). Courtney et al. (2009) also found that poorer health status hampered residents’ social engagement and perceptions of spiritual well-being. Connections with family and friends may also be disturbed, resulting in a loss of significant relationships (Knight & Mellor, 2007). These challenges and resulting losses have been linked to diminished quality of life (QoL) and psychological well-being (Drageset et al., 2011; Ercan-Sahin & Nuran Emiroglu, 2018). There have, however, been efforts to improve these conditions. Various studies have highlighted a shift from focusing solely on physical health to a more holistic approach (Hudson, 2012; Wright-St Clair et al., 2012). In other words, studies are shifting from a patient hood to a personhood perspective (Chochinov et al., 2015; Kong et al., 2017; Parkinson et al., 2020).

*Family Well-being*. Numerous studies have demonstrated that families remain involved in caring for relatives upon placement in LTC (Barken & Lowndes, 2018; Gaugler, 2005; Ryan & McKenna, 2015). Families support residents’ quality of life (McCabe et al., 2021; Shin & Park, 2018; Unsar et al., 2016). Other studies have explored the experiences of family members during the transition of a relative to long-term care. There is less research exploring the well-being of family members of residents. Evidence, however, indicates that the amount of time spent visiting the facility, changes in the caregiving role, dissatisfaction with care, challenges to the ability to communicate with staff, and functional decline of the resident all contribute to caregiver burden and distress (Bramble et al., 2009; Candy et al., 2015; Deborah, 2007; Schulz et al., 2004). Given that family members play an integral part in a resident’s care, this study explored bereaved family perceptions of care for their relatives during Covid-19. Bereaved family perceptions of care delivered to relatives during their final illness are regarded as an important indicator of the quality of that care (Teno, 2005).

*Well-being of LTC residents amidst Covid-19.* Given their age and comorbidities, older people in long-term care have a greater risk of death from Covid-19 (Comas-Herrera et al., 2020). The risks of rapid and silent spread of the infection from Covid-19 are further increased by the lack of capacity for social distancing from staff and other residents (Médecins Sans Frontières (Doctors Without Borders), 2020). To prevent and control Covid-19 infections, LTC facilities internationally have instituted restrictive measures, including a prohibition on visitors (Wang et al., 2020). These restrictive measures have severe implications for residents’ well-being. Older adults in long-term care are more vulnerable to social isolation and disconnection (Santini et al., 2020). An integrative review by Hugelius et al. (2021) exploring the effects of visitor restrictions in healthcare settings (including long-term care) found that imposed restrictions increased patient loneliness, depressive symptoms, agitation, aggression, and decreased cognitive ability and overall dissatisfaction. Family members experience increased worry, anxiety, uncertainty, and a heightened need for information from care providers (Hugelius et al., 2021).

*Aotearoa/New Zealand*, like other industrialised societies, has an ageing population. Persons over 65 constitute 15% of the population (Statistics New Zealand, 2019b). This number is anticipated to increase to 23.1% by 2041 (Statistics New Zealand, 2016). The number of adults over 85 years is also rising rapidly. The number of people over 85 is expected to triple in New Zealand by 2043 (260,600 people or 4.4% of the population)(Statistics New Zealand, 2016). With an increase in older adults in New Zealand society, there has been a concomitant increase in demand for long-term care (LTC) facilities. In New Zealand, two-thirds of adults reaching 85 years of age will eventually reside in an LTC facility (Broad et al., 2015). LTC facilities provide housing and support for persons whose care needs cannot be met within the home (Holdaway et al., 2021). Currently, LTC in New Zealand is provided by public, private, and religiously-based organisations (Thornton, 2010). LTC facilities are defined based on the level of dependency: ‘hospitals’ (24-hour nursing or medical care), ‘rest homes’ (support, but not 24-hour nursing or medical services), dementia (secure environment), and psychogeriatric (for residents with more challenging behaviours who require specialist nursing care) (Ministry of Health New Zealand, 2019).

Although New Zealand is justifiably cited for its national elimination strategy (Jefferies et al., 2020), the pandemic has significantly impacted older adults, particularly those in LTC. New Zealand recorded the first clusters of COVID-19 at the end of March 2020, followed by a six-week national ‘lockdown’ period (Moir, Lesa, & Ritchie, 2021). Despite infection control measures, including restrictions on visitors (Health Quality and Safety Commission (NZ), 2020), 16 of the 22 recorded deaths from COVID-19 as of November 2020 were persons in LTC facilities (Jeffries et al. 2020). Given the large number of older adults and families who will experience life in New Zealand LTC facilities in the era of COVID-19, it is important to explore what constitutes well-being within this setting.

*Long-term care models of psychosocial well-being.* Traditional biomedical models have focussed on a curative and individualist approach to supporting health in older adults(Frey, Powell, & Gott, 2013). Both hedonic (positive affect and life satisfaction) (Kahneman et al., 1999) and eudemonic (sense of personal growth) (Waterman, 2008) models frame well-being in terms of the individual. Currently there has been a shift in emphasis from a biomedical to a holistic approach to health (Frey et al., 2013; Huber et al., 2016; Wade & Halligan, 2017), including LTC (Abbate, 2021). Several models of care, “culture change models” (Rahman & Schnelle, 2008), have been widely implemented in industrialised societies, including the Eden Alternative, the Green House Project, Wellspring, and the Household Model (Hill et al., 2011; Shier et al., 2014). Although culture change models vary in practice, the common principles incorporate resident-directed care, a homey environment, close relationships, staff support and empowerment, non-hierarchical decision-making, and quality improvement systems (Koren, 2010). However, although these models are more inclusive, there remains an emphasis on autonomy and self-determination.

In Aotearoa/New Zealand, a history of challenges for Māori accessing and receiving healthcare delivered within a biomedical worldview has further compounded historical health inequities between Māori and NZ Europeans (Durie, 2005, 2006). The ‘Whare Tapa Whā’ model was introduced to address the need for culturally appropriate care (Durie, 1985). The model is widely utilised in New Zealand as a preferred definition of health (Durie, 1994; King, 2000). It is included in all healthcare professional training or hospital and community settings (Lambie et al., 2015). The ‘Whare Tapa Whā’ model draws on the analogy of the meeting house (Durie, 1994). The four sides represent the four dimensions of well-being: *te taha wairua*, the spiritual dimension; *te taha hinengaro*, the emotional aspect; t*e taha tinana*, the physical body; and *te taha whānau*, the family/extended family and the broader community (Durie, 1994, 2005, 2006). Balance among the four dimensions is required to enjoy well-being (Durie, 1994). It is important that a model incorporating Māori’s unique perspective of health be utilised to guide improvement in care in New Zealand society. It is proposed that the holism expressed in the ‘Whāre Tapa Whā’ model could be utilised as a framework for exploring what constitutes well-being for Māori and all other cultures in New Zealand. Previous research by Moeke-Maxwell et al. (2020) has extended the model (*Whare Tapa Whā Older Person’s Palliative Care Model*) in a study of family carers (including Māori carers) to inform improved end-of-life care for persons in advanced age. However, the Whare Tapa Whā health model has not been specifically extended as a framework to address the psychosocial needs of LTC residents and their families. Drawing on the Whare Tapa Whā health model, the goal is to link a model of well-being with the experiences of well-being for residents and bereaved family members. This is particularly important given the new reality imposed by the ongoing threat of COVID-19. The following questions guide the research:

- What are the components of well-being for LTC residents and bereaved families?
- How did the experience of Covid-19 restrictions shape the well-being of LTC residents and bereaved families?
- How does the Whare Tapa Whā health model relate to well-being constituents experienced by residents and bereaved family members?

## Design and Method

### Design

A qualitative descriptive design was employed. Five residents and six family members of previous residents were recruited from one LTC in one urban centre. The total number of interviews was influenced partly by the construct of saturation (Charmaz, 1990) and partly by the New Zealand COVID-19 alert level restrictions at the time of data collection. The selected LTC facility had participated in the Supportive Hospice and Aged Residential Exchange (SHARE) intervention, designed to enhance aged residential care staff’s palliative care skills. Components of the intervention included clinical coaching by a palliative care nurse specialist, role modelling of advance care planning conversations, and debriefing of LTC staff following a resident death. The intervention, implemented over one year (2019), is described in a separate article (Frey, Boyd, Robinson, Foster, & Gott, 2017). The purpose of the post-intervention interviews conducted in 2020 was to explore residents’ and families’ unmet psychosocial and spiritual needs as identified during the larger evaluation.

### Recruitment

#### Residents

The ability of residents to give fully informed consent was identified by clinical staff. Only those residents who could provide fully informed consent were recruited to participate in this study.

*Bereaved family members* were recruited by the LTC manager, who passed on the contact details of the interested bereaved family to the researchers. The bereaved families, rather than current family members, were chosen for interviews as there were no ethical issues around comments impacting the care of their relatives. There are no definitive guidelines for determining the optimum time frame for acquiring information from next-of-kin. Although it has been suggested that research conducted during the first six months of the bereavement period is most conducive to narrative reconstruction (Williams, Woodby, Bailey, & Burgio, 2008), sufficient after-death time needs to be allocated to reduce respondent vulnerability to harm and protect their emotional well-being (Rejnö, Danielson, & Berg, 2013). For that reason, bereaved families were selected who had a relative who died in the facility 12 months after the implementation of the educational intervention. Interviews were undertaken between July and September 2020. A full description of the sample and recruitment is supplied in an earlier article (Frey & Balmer, 2021).

The five resident participants (two women, three men) were most often over the age of 60 years of age (four) and of Pacific ethnicity (two). The remainder identified as either European (one), Asian (one), or other (one). The si bereaved family members were most often female (four). The majority (five) were under 80 years of age. The bereaved family most often identified as Maori (three), while the remainder reported both NZ European (one) and Māori (one), Pacific (one), or East Asian ethnicity (one) (Table 1).

**Table 1:** Demographic characteristics of residents and bereaved family members (n = 11)

	Frequency
**Resident**	5
Gender	
*Male*	3
*Female*	2
Age	
*Lowest to 60*	1
*61–89*	2
*90 and over*	2
Ethnicity	
*European*	1
*Pacific*	2
*Asian*	1
*Other*	1
Religion	
*Christian*	3
*Catholic*	1
*Other*	1
**Bereaved Family**	6
Gender	
*Male*	2
*Female*	4
Age	
*< 60*	2
*61–80*	3
*81+*	1
Ethnicity	
*NZ European*	1
*Maori*	3
*Asian/East Asian*	1
*Pacific*	1
Religion	
*Christian*	3
*Catholic*	1
*Other*	2
Relationship	
*Child*	3
*In-law*	1
*Grandchild*	1
*Partner*	1

### Interview Guide

A semi-structured interview guide was utilised. The interview guide was developed from a review of the literature. The purpose of the guide was to investigate resident and bereaved family spiritual and psychosocial needs and participant views of how best to meet those needs. The guides included a subsection with open‐ended questions to solicit the participants’ perceptions of quality of life and well-being. Topics included perceptions of loneliness or isolation during Covid-19 lockdowns and participant views on improving mental health and social well-being (Suppl 1 and Suppl 2). Questions focussed on perceptions of the current quality of life and well-being, changes in well-being since moving to LTC, and the impact of COVID-19.

### Ethical Considerations

The facility’s clinical staff determined LTC resident participants’ competence to consent to participate in the study. The research obtained informed written consent from all participants. This involved a process of distributing information about the study, including the research funder, aims and objectives, how the findings would be used, the time and activities involved, and how the data would be recorded, stored, and destroyed. Participants then had the opportunity to consider their participation and choose whether to opt in. Participants had the right to withdraw themselves or their data up to November 2019. Limited demographic data have been presented to protect participant identities. This limitation is of particular importance concerning indigenous data given the history of the exploitation of indigenous knowledge and the lack of collective rights and privacy within existing Western data environments (George et al., 2020; Kukutai & Cormack, 2020; Rainie et al., 2019; Walter & Suina, 2019; West et al., 2020). The study was approved by a New Zealand regional health ethics committee.

### Analysis

Studies have examined resident experiences during Covid-19 (Chee, 2020; Siette et al., 2021) and the experiences of bereaved relatives of residents who died during Covid-19, e.g. (Collier et al., 2023; Hack et al., 2022; Yildiz et al., 2022). Fewer studies have examined well-being during Covid-19 from multiple perspectives. Comparison is central to qualitative analysis (Tesch, 2013). Using the constant comparative method outlined by Boeije (2002), interviews with residents and bereaved families were compared in respect to the experience of a specific phenomenon (Covid-19). The analysis integrated both inductive and deductive approaches. In this instance, thematic analysis was utilised. Thematic analysis identifies patterns in data, such as commonalities and differences across cases (Braun & Clarke, 2006). The researchers independently coded the resident and family data. These separate analyses were then crosschecked to uncover related and/or deviant cases/themes and to develop an overall interpretation of the data. In each dataset, open codes were used to identify all instances where residents and bereaved families implicitly or explicitly referred to aspects related to subjective well-being as defined above. The researchers, working independently, developed data-driven codes related to the meaning of well-being. Codes were subsumed within larger themes. Themes were then linked with dimensions of the Whare Tapa Whā model (Durie, 1994). Using theory‐driven codes can alert researchers to concepts and processes that may not be identified through inductive processes alone (MacFarlane & O’Reilly-de Brún, 2011).

### Findings

We explored resident and bereaved family participants’ perspectives of well-being in LTC. Themes ***(****in italics*) developed from all twelve interviews were linked to the corresponding dimension of the framework of the *Whare Tapa Whā’* model (**bold**). Findings were divided into two groups (residents and bereaved family members) to compare perspectives. Table 2 (residents) and 3 (bereaved family) portray themes, definitions, and their relationship to the model. Both success and failure in achieving psychological well-being were evident in the data.

**Table 2:** Resident Wellbeing

Dimension	Definition	Theme	Quote
Whānau/Family Domain	family/taha whānau (social wellbeing)	Connection	Yeah. I’ve got a big family, my dear. (Mr L)
Mental/Emotional Domain	mental or emotional health/taha hinengaro	Psychosocial Wellbeing	I’m not lonely, I was busy and making the music, watch the TV. (Ms G)
Spiritual Domain	spiritual health/taha wairua (sense of meaning and purpose)	Spiritual Practice	I’ve been a Christian, what, well, I don’t know, 70 years, 80 years. 60, 70 years. (Mr K)
Physical Health Domain	physical health/taha tinana	Physical Wellbeing	Well, I had a stroke and I went to the hospital and they treated me. Once I was sort of stabilised, I was sent here because I can’t walk. (Ms F).

**Table 3:** Bereaved Family Wellbeing

Dimension	Definition	Theme	Quote
Whānau/Family Domain	family/taha whānau (social well-being)	Connection	Oh, look, he would have his friends come in and play the guitar to him and we had this little tape deck with Samoan songs on it. (Ms Z)
Mental/Emotional Domain	mental or emotional health/taha hinengaro	Psychosocial Wellbeing	I’m not a person that’s good with death by any means (Ms H)
Spiritual Domain	spiritual health/taha wairua (sense of meaning and purpose)	Spiritual Practice	I did ask them to make sure she could get there, but they told me that whenever they said, you know, there’s a church service on, would you like to go, she always said no.(Ms E)
Physical Health Domain	physical health/taha tinana	Physical Wellbeing	I’ve been given a diagnosis of chronic obstructive lung disease which I don’t embrace (Ms A)

#### Whānau/Family Domain

*Theme – Connection* was experienced as a sense of continuity and social support. This connection’s absence or potential absence could produce feelings of loneliness and social exclusion.

*Family -* Families play a key role in residents maintaining feelings of social inclusion and an overall sense of belonging, thus reducing the consequences of social exclusion. This role could be experienced both as a benefit and a burden. Ms. Z recalled:

*I was instrumental in getting her [sister] to visit her [mom] before she passed and spent some quality time with her…and she [sister] came with her two grown-up sons, and we had a little family reunion here. We had a very happy time then, and she enjoyed it very well with them.* (Ms. Z)

Ms. H expressed relief that her father had died before lockdown restrictions came into force. She was grateful that her father had not experienced the psychological distress of separation. She remarked:

*Oh, you know, he would’ve been really; he couldn’t have handled it. I would’ve almost felt that I had to go into lockdown with him. I would, to be honest, I would either have had to bring him back home or maybe go into lockdown.*

*Residents -* COVID-19 restrictions also affected the ability to connect and consequently influenced psychosocial well-being. For some resident participants, COVID-19 restrictions affected their enjoyment of life. Ms. F stated the following:

*Yeah, you’re telling me. I’ve been locked up in here for weeks on end, two lots. I’ve just come out now.*

***Interviewer. You think since you’ve been here that your quality of life, like in the last year,. .. has gone down or up?***

*Down. Down. Yeah… I don’t want to do anything now*.

In contrast, Ms. G felt that Covid-19 restrictions had little impact on her connection with others. She stated:‘ *No, no. I’m not lonely; I was busy and making the music, watch the TV … my life has kept moving”*. (Ms. G)

#### Mental/Emotional Domain

Theme – Psychosocial Well-being- The challenges imposed by the Covid-19 pandemic had significant implications for residents’ and their families’ psychological and emotional well-being. These impacts extended beyond the effects of public health restrictions.

*Family* - The death of a relative affects mental and emotional well-being. Mrs. A found that the death of her husband’s mother had a dramatic impact.

*I felt that after my husband’s mother died, you know, the funeral was within two days. And I came back absolutely devastated. It was just like her life was all wrapped up, and that was it. (Ms. A)*

*Residents* – The additional pressures on staff meant that the emotional needs of residents were often not addressed. When residents’ emotional needs were addressed, they perceived themselves as ‘all right.‘ However, Mr. K saw the emotional aspect of care within LTC as lacking.

**Interviewer. Within this Facility, do you have any Other Emotional Support here?**

*No. No, I mean, the nurses are very good, but you know, that’s sort of practical and not emotional, you know?* (Mr. K)

For one resident, Covid-19 was reported to be a source of racist remarks from others, based on the presumed country of origin of the virus.

*Hell, yeah, I want some peace and quiet. Whenever I travel from here past anybody, the big sound will be like a foghorn warning; here comes COVID-19, COVID-19.*

(Mr. L)

In contrast, Ms. B described her life within LTC during COVID restrictions as “all right.“ She reported utilising television to fill her time.

*I find that the TV takes up my time because I can change channels if I want to. No, I just sit here and watch TV; that’s about all that’s different.*

*Oh yes, it’s all right.* (Ms. B)

#### Spiritual Domain

Theme Spiritual Practice – spirituality was experienced as a challenge and support based on perspective. For families, spiritual challenges surrounded the ability to meet a perceived need, while spirituality in the form of religious belief was experienced as a source of comfort for residents.

*Family* - Family members wanted to ensure spiritual care was provided for their relatives. However, sometimes logistical challenges or the culture of the facility prevented this. Ms. E stated:

*Well, I’m quite religious... So yeah, but I kind of felt that that was sort of a big gap which I kind of wished I could fill… things like the service, there were services available. But they were, for someone of her, extremely limited... somehow get her, you know, upstairs in elevators and, you know, to a different place. And she just, it was just too much to contemplate. (Ms. E.)*

Mrs. G spoke of how spiritual support was provided “behind the scenes”:

*It just seemed to me that we needed that spiritual input at that point and that that would be meaningful…the nursing staff were from the Philippines, and it was meaningful to them. So actually, what happened behind the scenes is they began to pray with him. (Mrs. G)*

*Residents*- Spiritual needs of residents were sometimes used as a source of solace to deal with physical challenges. Mr. K reflects on the following:

*As a Christian, you look at it all, and you say, well, where’s God? What’s he done? Why am I like this? He’s supposed to be looking after me. But then, ‘cause I know quite a few other Christians have got cancer, they’ve got this, they’ve got that…and it’s just part of the deal.*

(Mr. K)

#### Physical Health Domain

Physical Well-being- Families more often spoke of the pandemic’s personal or potential impact on their physical welfare. In contrast, residents spoke of physical health issues as an expected outcome of advanced age.

*Family***-** While none of the resident participants were directly impacted, one family member reported the effect of COVID on her physical health. Ms. F explained:

*Yes. I picked up this terrible virus, and I was tested, and it was really quite hard being on my own, especially at night when you’re really ill…and that was quite difficult, yeah, mmm. I know, it was terrible. (Ms. F)*

In contrast, Ms. C spoke of gratitude for being in New Zealand and the physical protection that offered:

*I was fortunate that in being here in New Zealand because we enjoyed relative more freedom in every way than people in India. So that way I was really happy…I didn’t feel much different from the life that I used to live normally in the Covid circumstances, except that the movements were restricted. (Ms. Z)*

*Residents -*. For residents, a decline in physical health was expected and spoken about matter-of-factly. Residents often related their illnesses to their arrival in LTC. Ms. F stated

*I had a stroke, and I went to the hospital, and they treated me. Once I was sort of stabilised, I was sent here because I can’t walk. My left side was, you know, I’m stuck with my left arm and leg, arm recovered, not my leg*..

### Interconnections

The Whare Tapa Wha model recognises four interconnected, interacting aspects of health. These interconnections were evident in the data. For example, Mr. K linked physical health (*te taha tinana*) and spiritual beliefs (*te taha wairua)*,

*My physical quality is not the same, obviously, you know? I can’t walk, have to stay here. Can’t live at home, can’t live in a flat like I was. But my spiritual quality is 100%*, (Mr. K)

Ms. H linked mental well-being (*taha hinengaro*) with family connection (*taha whanau*). She spoke about the loss of her parents and the emotional impact:

*And although it was hard when I lost her…it was worse when I lost him because both parents have gone. So when she died, you still had him. And what surprised me ‘cause although her death prepared me for losing a parent, it was worse than I thought it would be.* (Ms. H)

## Discussion

The COVID-19 pandemic has caused heated discussion around the impact of care models in LTC facilities during the pandemic. Internationally, COVID-19 infection control response measures adversely affected culture-change practices in LTC homes. Care shifted from a person-centered to an institutionalised model of care with negative consequences for residents and families (Iyamu et al., 2022). Yet, international evidence indicates that LTC facilities that moved farthest from the medical model of LTC had reduced mortality and morbidity and more psychosocial benefits (Power & Carson, 2022). However, these studies primarily focused on specific aspects of care and the impact of Covid-19 restrictions (Li, 2021; Tandan et al., 2023; Young et al., 2023; Zimmerman et al., 2021) Evidence from the current study indicate that Te Whare Tapa Whā can be applied to describe the total well-being of the resident and their family/whānau. Our study adds to the scarce research utilising an integrated well-being model, which aligns with the experiences and conditions of New Zealand older adults in LTC and their families during the pandemic (Cheung et al., 2022). New Zealand society is becoming increasingly diverse (Māori (16.5% of the population), Pacific (9.1%), and Asian (15.1%) (Statistics New Zealand, 2019a). Over 27% of people counted in the 2018 census were not born in New Zealand (Statistics New Zealand, 2019a). An indigenous model of health such as Whare Tapa Whā’ can have utility in describing the psychosocial needs of older Māori and other New Zealand ethnicities now and into the future. Findings indicate a resonance between the model domains and themes as outlined below:

### Family/Whanau Connections

The recent pandemic has provided a challenge highlighting the gaps in well-being for LTC residents and families. COVID-19 has created uncertainty and emphasised the need for balance to maintain well-being. Either directly or indirectly, COVID-19 and its effects on the well-being of residents and family members underlay many participants’ experiences. In line with other current research (Simard & Volicer, 2020), separation from family due to Covid-19 restrictions depressed residents’ perceptions of well-being. Policies shaped by a biomedical model often prioritise infection control over the physical and psychosocial benefits provided to residents from the family connection (Kemp, 2020). Although unable to replace physical contact with friends and family, technology can give residents a sense of normalcy and continuity in daily life.(Chu et al., 2021).

### Mental/Emotional Well-being

Negative impacts were identified by Samuels et al.‘s (2021) research with community-dwelling older adults. The authors found that television watching during COVID increased sedentary practices. Television could, however, be utilised to improve resident mental health. Research by Goodman-Casanova et al. (2020) conducted with LTC residents in Spain during COVID found that health programs delivered via television may provide cognitive stimulation (e.g., recreational activity, memory exercises as intellectual activity). While not specifically designed for LTC, in New Zealand, a free-to-view national television programme aired during the COVID lockdown targeted older adults, providing information on nutrition, sleep, home safety, mental well-being, and maintaining social links (Parsons et al., 2021).

### Spirituality and Spiritual Well-being

Previous research has identified that a lack of attention to spiritual needs makes patients and their families feel unsupported (Carlson, 2007). The current study demonstrates that this lack of support was exacerbated by the restrictions imposed due to Covid-19. Yet spirituality is a key aspect of ageing. Older adults are more disposed toward spiritual beliefs when compared to younger adults (Lowis et al., 2009; Stefanaki et al., 2014). Psychological, social, and religious support can provide a source of meaning for older adults (Washington et al., 2009).

### Physical Well-being

Family relationships are an important factor in determining the health and well-being of residents, particularly among Māori and Pacific Peoples (Durie, 1994; Pulotu-Endemann & Tuʼitahi, 2009). However, in contrast to research by Ham et al. (2021) examining bereaved families’ quality of life pre and post-Covid-19, the family members’ health in the current study suffered due to the pandemic, a finding also confirmed in other family members bereaved during the COVID-19 restrictions in New Zealand (Collier et al., 2023). This result aligns with research by Raker et al. (2020) examining the consequences of Hurricane Katrina, which concluded that there were indirect impacts on survivors’ mental and physical well-being through exposure to stress and potential trauma.

Research has indicated that the social and physical effects of changes to routine imposed to prevent the spread of infection during the pandemic could contribute to declines in physical welfare (Manderson & Levine, 2020). However, in line with research by Cheung et al. (2022), residents in the current study reported no ill effects of Covid-19 restrictions on aspects of their physical health as identified by the model. Longer-term effects of the COVID-19 pandemic on LTC populations should also be investigated.

### Strengths and Limitations

This research explored the well-being of LTC older adults from a holistic point of view—one that addresses multiple dimensions of a person’s life, such as those included in the four major life domains and indicators of each domain, as well as presenting perceptions from more than one source, such as older individuals as well as family members. The research also adds to the body of knowledge (e.g., Kaelen et al., 2021; O’Caoimh et al., 2020; Tupper et al., 2020) conducted during the first Covid-19 wave on how residents perceive and experience well-being in light of prevention measures taken for them by the LTC facilities. Although this study sought to expand the application of Whare Tapa Whā’ to all LTC residents, one study limitation is the absence of the voices of more Māori and ethnic minorities in advanced age themselves. Future research could explore the model with them and their family members together. In addition, as a follow-up study, sampling was necessarily hampered by a limited participant pool and therefore findings may not be indicative of wider New Zealand LTC population and their families.

### Implications and Recommendations

Despite the pandemic, life residential aged care must that provide environments that offer meaning to residents’ lives (Carlson et al., 2009). Residents should be included in the decision-making processes and made to feel needed and included, particularly within the context of COVID-19 (Anderson et al., 2020). As the Whare Tapa Whā model indicates, a holistic approach incorporating the four domains is required for well-being. Health professionals’ capability to provide holistic care, rather than focusing on the physical, would benefit older people and their families. Drawing on the Whare Tapa Wha model, future research conducted with residents’ families and staff should explore the most effective tools to meet the psychosocial needs of residents (Amabile, 2019). Additionally, given the ongoing challenges of the pandemic, further research into the long-term consequences of physical and social distancing on the well-being of LTC older adults and their families is needed.

## Conclusion

Study findings indicate that the provision of well-being for older adults and families in LTC must give attention to an older person’s relational, cultural, and spiritual needs as well as physical health. This will enhance the foundational and interconnected pillars of holistic care. The challenges of COVID-19 further highlight the need for an integrated approach to assessing risks to psychosocial well-being while safeguarding the physical health of LTC residents (and their families).

## Data Availability

Due to the nature of this research, participants did not agree for their data to be shared publicly, so supporting data is not available.
